# Enhancing uterine contraction detection through novel EHG signal processing: a pilot study leveraging the relationship between slow and fast wave components to improve signal quality and noise resilience

**DOI:** 10.3389/fphys.2025.1568919

**Published:** 2025-05-27

**Authors:** Hansong Gao, Zichao Wen, Meng Jiang, Yuan Nan, Yong Wang

**Affiliations:** ^1^ Department of Electrical and Systems Engineering, Washington University, St. Louis, MO, United States; ^2^ Department of Obstetrics and Gynecology, Washington University School of Medicine, St. Louis, MO, United States; ^3^ Department of Biomedical Engineering, Washington University, St. Louis, MO, United States; ^4^ Mallinckrodt Institute of Radiology, Washington University School of Medicine, St. Louis, MO, United States

**Keywords:** Electrohysterography, signal enhancement, uterine contraction, slow wave, uterine signaling

## Abstract

Uterine contractions, driven by complex electrical activities within the uterine smooth muscle cells, play a critical role in labor and delivery. Various techniques, including EHG and EMMI, have been developed to record and image uterine electrical activities. Both EHG and EMMI use a bandpass filter (fast wave 0.34–1Hz) to preserve uterine contraction activities. However, high-frequency signals are usually weak and are prone to multiple sources of noise and artifacts, significantly impacting the accuracy of contraction detection and subsequent analysis of long- and short-distance signaling in the laboring uterus. Existing methods, such as Zero-Crossing-Rate (ZCR) and Teager-Kaiser Energy Operator (TKEO), employ the transformation of fast wave signals to detect uterine contractions and are still limited by the EHG signal quality. This work proposed a novel method that combines high-frequency (fast wave, 0.34–1Hz) and low-frequency (slow wave, 0.01–0.1Hz) components of uterine electrical signals to generate enhanced EHG signals. Incorporating slow-wave signals offers additional information rather than relying solely on fast wave signals like ZCR and TKEO. Our approach utilizes the stability of slow wave signals to enhance the more noise-prone fast wave signals. This method significantly improves the quality of uterine contraction detection, as evidenced by enhanced signal contrast between contractions and baseline activity. The improved signals enable more accurate detection of contractions and more detailed spatial analysis of uterine contraction propagation. This signal enhancement technique holds great potential for advancing the understanding of long- and short-distance signaling during labor, paving the way for more precise labor management and better maternal-fetal outcomes.

## 1 Introduction

Rhythmic uterine contractions (Cohen, 2017; Young, 2015) of labor are produced by the activation and relaxation of the uterine smooth muscle myocytes (Young, 2007; Young and Barendse, 2014). These cellular electrical activations are driven by calcium metabolism, which is a complex network of ion channels, pumps, and exchangers (Garfield et al., 2020; 2005; Garfield and Maner, 2007; Lammers, 2013). Collectively, millions of myocytes are composed of the myometrium, and the electrical signals of myocytes contribute to the electrical signals that are observable and measurable on the body surface to reflect the uterine activities noninvasively (Rosen and Yogev, 2023). Improving our understanding of the relationship between these electrical signals and the effectiveness of uterine contraction to advance labor will enhance our ability to clinically manage both term and preterm labor (Li et al., 2022; Romero-Morales et al., 2023).

Various techniques have been developed to record and analyze uterine electrical activity. Uterine electromyography, also called electrohysterography (EHG) (Devedeux et al., 1993; Jacod et al., 2010; Larks and Dasgupta, 1958), is a non-invasive method employing surface electrodes on the abdominal wall and has emerged as a promising tool for monitoring uterine contractions (Euliano et al., 2009; Jacod et al., 2010; Muszynski et al., 2018) throughout gestation and during labor. This technique offers advantages over traditional tocodynamometry (EULIANO et al., 2013), including improved sensitivity and the ability to detect contractions earlier in pregnancy. More recently, advanced techniques such as electromyometrial imaging (EMMI) (Wang et al., 2023; 2020a; 2020b; Wang and Wang, 2020; Wu et al., 2019) have been developed, combining high-density surface electrode arrays with magnetic resonance imaging (MRI) to create detailed three-dimensional maps of uterine electrical propagation. These techniques provide more comprehensive data for a better understanding of long- and short-distance signaling in the laboring uterus and facilitate clinical decision-making.

EHG and EMMI employ band-pass filtering of 0.34–1 Hz to preserve the signal of uterine activities. Traditional energy-based contraction detection methods perform well in detecting uterine contractions from EHG when the signal-to-noise ratio (SNR) is sufficient. However, the recording environment in the labor-delivery room is typically electrically noisy, and it is challenging for laboring women to stay static during the recording. Under such a noisy environment, EHG signals are very susceptible to low SNR and motion artifacts (Allahem and Sampalli, 2020; Chen et al., 2024a; Esgalhado et al., 2020; Ye-Lin et al., 2014). In EMMI studies, multi-channel abdominal EHG signals are often contaminated by various noise sources, including motion artifacts, power line interference, and crosstalk from adjacent muscles (Boyer et al., 2023). Low-quality signals increase the false positives (detecting contractions that are not occurring) or false negatives (missing real contractions) for uterine contraction detection. Such detection inaccuracy negatively impacts the accurate identification and analysis of potential EHG biomarkers for preterm labor or other pregnancy-related conditions (Marinescu et al., 2022). For example, previous studies have failed to pinpoint the onset of contractions or identify consistent directions of propagation using EHG or magnetometers (Young et al., 2023).

Conventional uterine contraction detection methods, such as energy-based approaches, can be easily affected by subtle electrical activities, including motion artifacts. One way to improve the performance of uterine contraction detection from EHG signals is to develop more sophisticated signal processing algorithms. Existing methods, including Zero-Crossing-Rate (ZCR) (Song et al., 2021) and Teager-Kaiser Energy Operator (TKEO) (Solnik et al., 2010; Vasist et al., 2022) are developed to increase uterine contraction detection accuracy. ZCR quantifies the frequency at which a signal transitions between positive and negative values or *vice versa*. ZCR is inherently linked to the frequency content and is an effective parameter for distinguishing between contraction and baseline noise segments. TKEO is a non-linear technique utilized to assess the instantaneous energy variations of the signal. By incorporating both amplitude and frequency components, TKEO enhances the accuracy of detecting the onset of uterine contractions. However, both methods are based on the transformations of the fast wave signals (0.34–1 Hz) without incorporating extra information, and the SNR of EHG still limits their performances.

In this study, we proposed a novel signal enhancement method designed to increase the accuracy of uterine contraction detection by significantly improving the signal quality by incorporating additional physiological information. The signal enhancement method filters the contraction signal into two distinct components: a high-frequency component (fast wave 0.34–1 Hz) and a low-frequency component (slow wave 0.01-0.1 Hz). Both components have been reported as reliable electrical signals recorded by conventional EHG electrodes (Garfield et al., 2020). Our data suggests that uterine contractions are highly correlated with both slow and fast wave signals. Notably, slow waves have larger magnitudes (Medel et al., 2023) and demonstrate greater robustness against contamination from the measurement noises and motion artifacts than fast wave signals. By incorporating slow wave signals alongside fast wave signals, we gained additional reference information to better identify the occurrence of contractions, thereby enhancing the quality of the fast wave signals. This approach led to a significant improvement in overall signal clarity and reliability.

The primary goal of the signal enhancement method is to improve the signal quality, thereby benefiting post-processing tasks, such as uterine contraction detection, which will facilitate a better understanding of long- and short-distance signals of the laboring uterus based on multi-channel EHG recordings.

The rest of this paper is structured as follows. Section 2 presents a comprehensive description of our proposed signal enhancement method, metrics to quantify signal quality improvements, and statistical tools to determine the significance of the difference observed in the metrics. Additionally, we describe the contraction detection method, and the approach employed to analyze the uterine contraction propagation. Section 3 provides a detailed comparative analysis of signal quality between fast wave and enhanced signals. We then evaluate the impact of our signal enhancement technique on contraction detection performance. Additionally, spatial patterns of uterine contraction propagation based on fast wave signals and enhanced signals are compared. Section 4 concludes with our findings and a critical discussion of the implications for uterine contraction monitoring. We address the limitations of our signal enhancement method and explore potential applications for future research and development.

## 2 Materials and methods

### 2.1 Data collection

**FIGURE 1:**
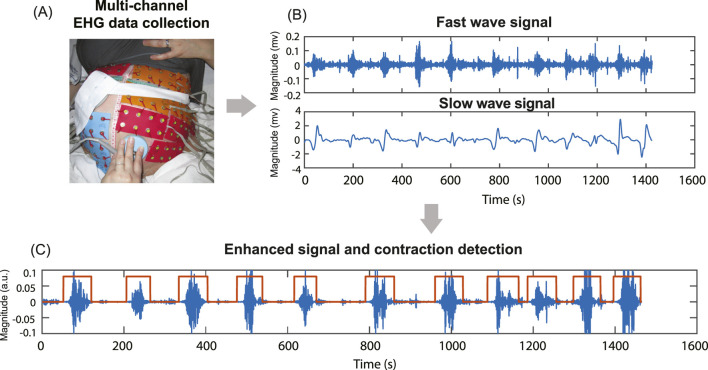
Multi-channel EHG data collection, filtering, signal enhancement, and contraction detection. **(A)** Multi-channel EHG signals were measured from up to 192 unipolar electrodes on the subject’s body surface. **(B)** The raw signal is filtered into fast (0.34–1Hz) and slow wave (0.01–0.1Hz) signals. **(C)** Signal enhancement combines fast and slow wave signals and contraction detection results based on enhanced signal.

This study utilized body surface multi-channel EHG data from five subjects in active labor with cervical dilation greater than 4 cm (Friedman and Cohen, 2023; Neal et al., 2010) at the time of data recording. The data were collected as part of the electromyometrial imaging (EMMI) study (Wang et al., 2023). For each subject, a multi-channel EHG recording, approximately 20 min in duration, was measured from up to 192 unipolar EHG electrodes placed directly on the body surface, targeting uterine contraction signals during labor, as shown in Figure 1. With unipolar recording, each channel was referenced to the average signal of the grounding channels. The temporal sampling rate for all recordings was set at 2048 Hz. The raw electrode recording was passed through a Butterworth filter with a frequency range (0.01–0.1Hz) to get the slow wave signals and a frequency range (0.34–1Hz) to get the fast wave signals. Channels with fast waves exhibiting a maximum magnitude greater than 0.3 mV or less than 0.01 mV were classified as low-quality channels and excluded from the computation. The entire signal processing pipeline was conducted using MATLAB (2022b). The procedure was approved by the Washington University Institutional Review Board (No. 201612140) and complied with its guidelines and regulations. The clinical characteristics of five subjects are shown in Table 1.

**TABLE 1:** Clinical characteristics of participants.

Clinical characteristics	Subject #1	Subject #2	Subject #3	Subject #4	Subject #5
Age	24	30	18	21	32
Delivery history	Multiparous	Nulliparous	Nulliparous	Multiparous	Nulliparous
Labor type	Induction	Induction	Induction	Induction	Induction
Gestation	40 weeks 1 day	39 weeks 1 day	39 weeks 1 day	37 weeks 0 days	40 weeks 2 days
Cervical dilation	4.5 cm	6.5 cm	6.5 cm	5 cm	7.5 cm
Number of electrodes used	144	108	108	128	128
Number of high-quality channels	119	92	74	115	123

### 2.2 Signal enhancement method

**FIGURE 2:**
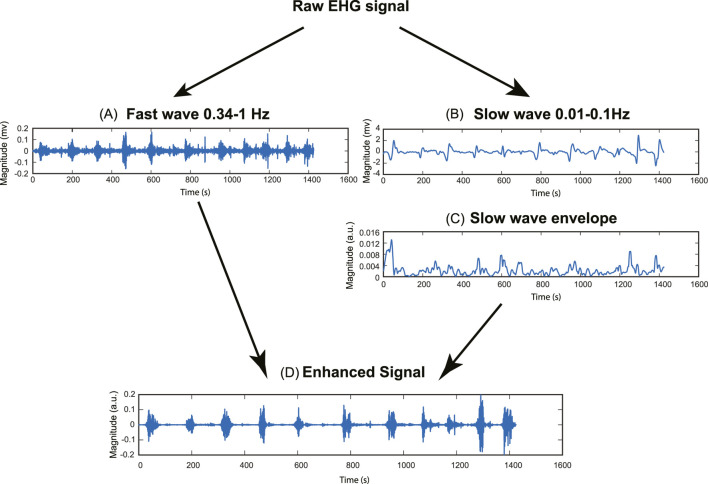
Pipeline for signal enhancement method. **(A)** Fast wave signal (0.34–1Hz). **(B)** Slow wave signal (0.01–0.1Hz). **(C)** Slow wave RMS envelope (Window size = 10s). **(D)** Enhanced signal.

A two-step signal enhancement method was developed to combine low-frequency signals and conventional uterine EHG signals, as shown in Figure 2. First, a fourth-order Butterworth bandpass filter of 0.01–0.1 Hz and 0.34–1 Hz was applied to extract the low-frequency EHG signal $s_{i}=s\left(t_{i}\right)$ and high-frequency EHG signal $f_{i}=f\left(t_{i}\right),i=1,2,\ldots ,N$ , respectively, where $s_{i}$ and $f_{i}$ are the voltage samples measured at the time $t_{i}$ . Without loss of information, the sampling rate of fast and slow wave signals was then down-sampled to 5 Hz. Next, the enhanced signal was defined by multiplying the high-frequency EHG signals by the root mean square envelope of the low-frequency signals (Equation 1).

$$en\left(t_{i}\right)={RMS}_{slow}\left(t_{i}\right)\cdot \;f\left(t_{i}\right)$$ (1)

The RMS envelope of the slow wave was calculated using Equation 2:

$${RMS}_{slow}\left(t_{i}\right)=\sqrt{\frac{1}{n_{i}}\sum_{j=\max\left(1,i-\frac{n}{2}\right)}^{j=\min\left(N,i+\frac{n}{2}\right)}{s\left(t_{j}\right)}^{2}}$$ (2)

where n represents the window length (n = 50). The term $n_{i}=\min\left(N,i+n/2\right)-\max\left(1,i-n/2\right)$ accounts for both full and partial window calculations. The latter is particularly relevant for the start and end portions of the signal, ensuring that the RMS is computed using the available data within the incomplete window segments.

The underlying rationale for improving the signal quality is to enhance the uterine contraction signal while suppressing the baseline noise signal. To achieve this goal, we used the root mean square (RMS) envelope of the slow wave as a weighting factor. This approach involves applying a higher weight factor to the contraction signal and a lower weight factor to the baseline noise signal, thereby increasing the contrast between the baseline noise and uterine contraction.

### 2.3 Evaluation metrics for signal quality

While the signal-to-noise ratio (SNR) is traditionally considered the most direct metric for assessing signal quality, its calculation requires precise knowledge of both signal and noise levels, which is challenging to obtain from our dataset. Instead of computing SNR, we implement three alternative metrics to address this limitation and provide a comprehensive evaluation of our signal enhancement method on the channel level. These metrics offer diverse perspectives on signal quality improvement, allowing for a robust assessment of the enhancement technique’s effectiveness. To describe the relationship between the metrics and signal quality, we first divide the EHG signal into subsequences using a sliding window operation and compute the energy for each subsequence. The energy of each subsequence is computed using Equation 3:

$$e=\sum_{1}^{n}{\left[i\left(t_{j}\right)\right]}^{2}$$ (3)

where *e* represents the energy of the input signal in a sliding window. n is the length of the sliding window ( $\left.n=50\right)$ . The input signal *i*, taken within a sliding window, can be either fast wave signals or enhanced signals.

**FIGURE 3:**
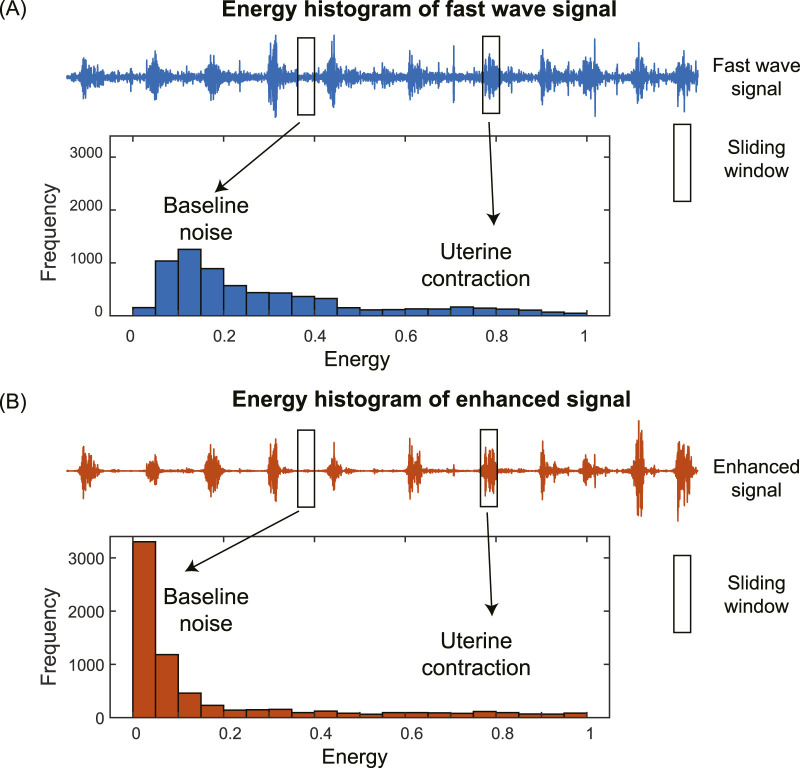
Energy histograms of fast wave signal **(A)** and enhanced signal **(B)**.

The histogram of the energies of subsequences in the EHG signal has two distinct components corresponding to baseline noise and uterine contraction, separately (Figure 3). For the fast wave signal, the two components are close to each other with low contrast, making them difficult to separate (Figure 3A). In contrast, for the enhanced signal, the two components are far apart with high contrast, making them easy to separate (Figure 3B). We used the following metrics to quantify the differences in the shapes of the histograms: skewness, kurtosis, and peak-to-average-ratio (PAER). To reduce the impact of spike artifacts, we used the 0.95 quantile of the data, as spike artifacts typically exhibit large magnitudes.

#### 2.3.1 Skewness

Skewness is a statistical measure that quantifies the asymmetry of a probability distribution. The skewness is computed using Equation 4:

$$Skewness=\frac{1}{N}\sum_{i=1}^{N}{\left[\frac{\left(e_{i}-\bar{e}\right)}{\sigma }\right]}^{3}$$ (4)

where $e_{i}$ represents the energy of the signal in a window, N is the length of the sliding window, $\bar{e}$ denotes the sample mean of the energy distribution, σ is the standard deviation of the energy distribution.

The skewness of the energy distribution of the enhanced signal is expected to increase compared to that of the fast wave signal. This is because the baseline noise is significantly reduced, causing it to shift to the left, resulting in a more positively skewed distribution, as shown in Figure 3.

#### 2.3.2 Kurtosis

Kurtosis is a higher-order statistical measure that quantifies the shape of a probability distribution, specifically focusing on the dispersion of data between the distribution’s center and tails. The kurtosis is computed using Equation 5:

$$Kurt=\frac{1}{N}\sum_{i=1}^{N}{\left[\frac{\left(e_{i}-\bar{e}\right)}{\sigma }\right]}^{4}$$ (5)

where $e_{i}$ represents the energy of the signal in a window, N is the length of the sliding window, $\bar{e}$ denotes the sample mean of the energy distribution, σ is the standard deviation of the energy distribution.

The kurtosis of the energy distribution of the enhanced signal is expected to increase compared to that of the fast wave signals. This is due to a significant reduction in baseline noise, causing it to shift to the left, leading to heavier tails and higher kurtosis, as shown in Figure 3.

#### 2.3.3 Peak-to-average-energy ratio (PAER)

Inspired by the Peak-to-noise ratio (PSNR) concept, we developed a new metric: Peak-to-average-energy ratio (PAER). It quantifies the ratio between the signal’s maximum energy and the average energy of the signal. We computed PAER using Equation 6:

$$PAER=10*\log_{10}\left(\frac{\max{\left(e\right)}^{2}}{mean{\left(e\right)}^{2}}\right)$$ (6)

where e represents the energies of subsequences.

For the enhanced signal, the baseline noise is significantly reduced, causing it to shift to the left and resulting in an overall decrease in the mean, as shown in Figure 3. The PAER is expected to increase since the maximum value of the energy distribution remains largely unchanged, and the mean value of the energy distribution decreases.

### 2.4 Evaluate the impact of signal enhancement on contraction detection

**FIGURE 4:**
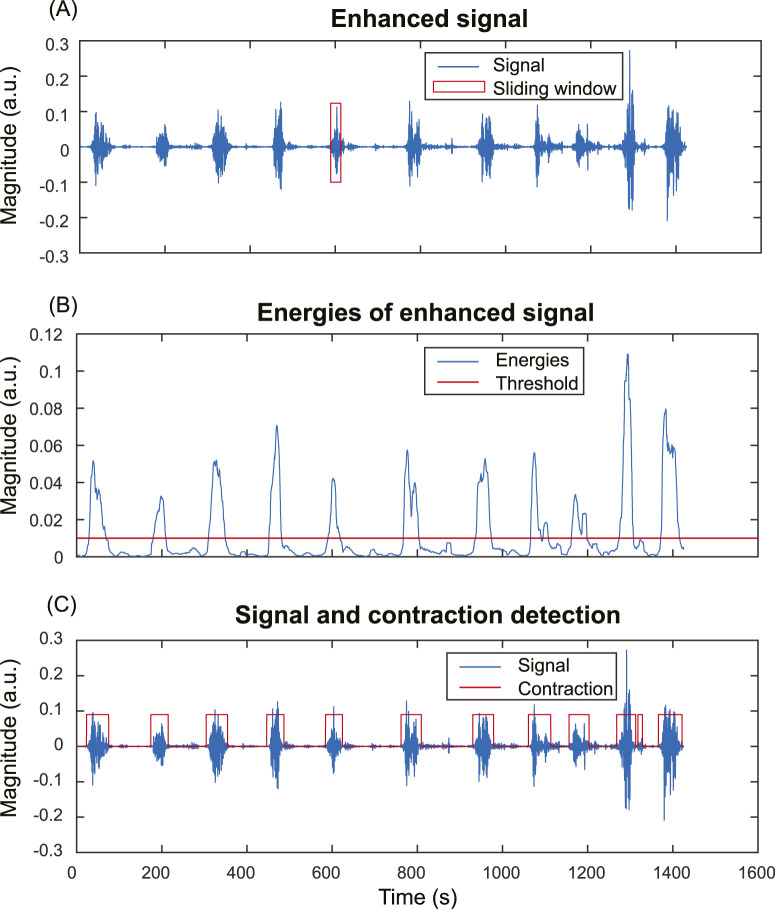
Energy-based uterine contraction detection method. **(A)** A sliding window operation is applied to compute the energy of each subsequence of the input signal. **(B)** Energies of the subsequences of the input signal. Each point in **(B)** corresponds to the energy of a subsequence in the sliding window in **(A)**. A threshold is applied to determine the uterine contraction and baseline regions. Energies higher than the threshold are identified as uterine contraction regions, while energies lower than the threshold are identified as baseline noise regions. **(C)** The input signal and uterine contraction detection results.

To evaluate the impact of signal enhancement on the accuracy of contraction detection, we compared the contraction detection results on fast wave and enhanced signals. Energy-based method (Horoba et al., 2001) is one of the most widely used methods in contraction detection. Figure 4 shows the pipeline of the contraction detection method using a single-channel enhanced signal as an example (Figure 4A). The energies of subsequences (Figure 4B) were computed for both fast wave and enhanced signals with a sliding window operation using Equation 3. A threshold is applied to determine the uterine contraction and baseline regions. The energies higher than the threshold were identified as uterine contraction regions, while energies lower than the threshold were identified as baseline noise regions (Figure 4C).

**FIGURE 5:**
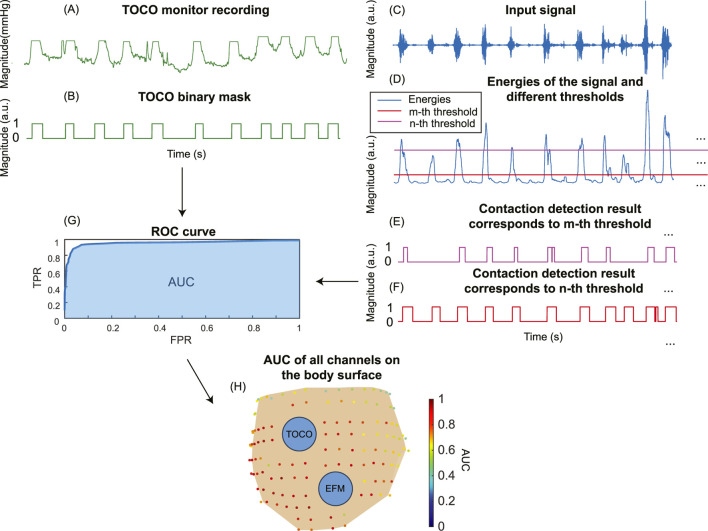
The pipeline to compute the ROC curve for TOCO and contraction detection binary masks. **(A)** Tocodynamometer (TOCO) recording. **(B)** TOCO binary mask. **(C)** Input signal. **(D)** Energies of the input signal are computed, and different thresholds are set to generate contraction detection masks **(E, F)**. **(G)** The ROC curve and AUC for a single channel are obtained by comparing the TOCO binary mask and contraction detection binary masks. **(H)** The AUC for all the channels on the subject’s body surface.

To demonstrate the improvement in contraction detection, a binary mask (Figure 5B) was generated to mark the timing of uterine contractions. First, we aligned the TOCO recording (Figure 5A) with the electrical signal (Figure 5C) by matching the start and end time points. Then, the binary mask is manually labeled by referring to the TOCO recording. The receiver operating characteristic (ROC) curve and area under the curve (AUC) (Figure 5G) were computed by comparing the TOCO binary mask (Figure 5B) and the predictions of different thresholds (Figures 5E,F). The thresholds for each point on the ROC curve are discretized by computing the mean value (μ) and the standard deviation (std) of the energies of the input signals (Figure 5D). These thresholds ranged from μ-3*std to μ+3*std, representing the data with z-scores from −3 to +3, which covers 99.7% of the data in a normal distribution. A step size of 0.05*std was used. The optimal threshold is defined as the one that maximizes the difference between true positives and false positives. The ROC and AUC were first evaluated using three representative channels, which included one high SNR fast wave and two low SNR fast wave channels. Subsequently, the AUC was computed for all the high-quality channels for each subject using the same pipeline (Figure 5H). These AUC results of all high-quality channels on the body surface are then used to evaluate the impact of enhanced signal on spatial signaling and propagation of the uterine contraction.

### 2.5 Evaluate the impact of signal enhancement on electrical signaling and propagation in the laboring uterus

**FIGURE 6:**
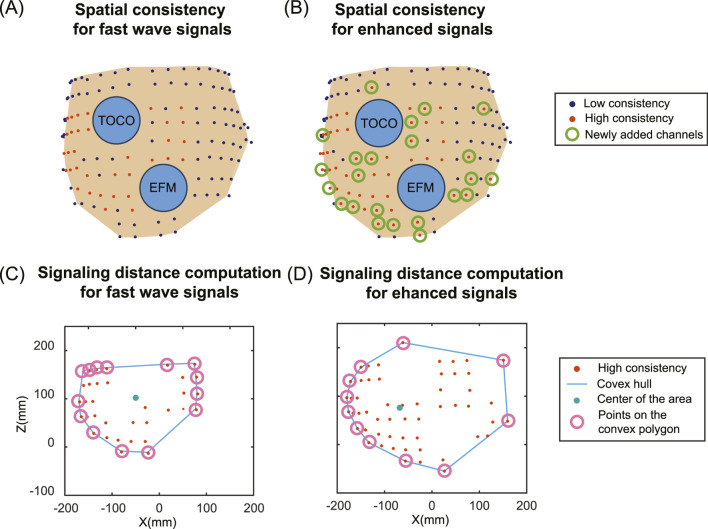
Spatial distribution of high consistency channels between multi-channel EHG and TOCO signals and signaling distance estimation for fast wave and enhanced signals. **(A)** Spatial pattern of the signal consistency for fast wave signals. **(B)** Spatial pattern of the signal consistency for enhanced signals. Deep blue dots indicate low consistency, red dots indicate high consistency, and green circles indicate newly added high consistency channels for enhanced signals compared to fast wave signals. Two blue circles represent the electronic fetal monitor (EFM) and tocodynamometer (TOCO). **(C)** The signaling distance computation for fast wave signals. **(D)** The signaling distance computation for enhanced signals. The red dots represent the high consistency channels. The blue lines represent the convex hulls. The green dots represent the geometric centers of the area, and the pink circles represent the points on the convex polygons.

To compare the impact on the signaling and propagation in the laboring uterus, we used AUC to evaluate the consistency between multi-channel EHG signals and the TOCO signal. Figure 6 shows the pipeline to compute spatial consistency and the estimation of uterine signaling using a representative subject (subject #5). We defined an AUC threshold of 0.8 (Çorbacıoğlu and Aksel, 2023) to identify high signal consistency with the TOCO recording (red dots in Figures 6A,B). To quantify the impact of enhanced signals on the spatial pattern of electrical activities in the laboring uterus, we compared the number of high consistency channels for enhanced signals (Figure 6A) to those for fast wave signals (Figure 6B) using Equation 7:

$$r_{c}=\left({ch}_{E}-{ch}_{f}\right)/{ch}_{f}$$ (7)

where ${ch}_{E}$ represents the channel number of enhanced signals exhibiting high consistency with the TOCO signal, ${ch}_{f}$ represents the channel number of the fast wave signals, which exhibit high consistency with the TOCO signal, $r_{c}$ represents the growth ratio of the channel number, comparing the fast wave and enhanced signals.

Based on the results of high consistency channels, we estimated the uterine signaling distance (Figures 6C,D). The locations of channels that exhibited high consistency are first projected along the y-axis (the direction from anterior to posterior). A convex hull is computed to enclose all the points, forming a convex polygon. The geometric center is computed by taking the mean value of the points on the convex polygon. The signaling distance d is estimated by computing the average distance between all the points on the convex polygon to the geometric center (Equation 8).

$$d=\frac{1}{n}\sum_{i=1}^{i=n}\sqrt{{\left(x_{i}-x_{c}\right)}^{2}+{\left(y_{i}-y_{c}\right)}^{2}+{\left(z_{i}-z_{c}\right)}^{2}}$$ (8)

where $x_{i},y_{i},z_{i}$ represent the coordinates of points on the convex polygon. $x_{c},y_{c},z_{c}$ represents the coordinates of the geometric center, and n represents the number of points on the convex polygon.

The growth ratio of the signaling distance is computed using Equation 9:

$$r_{d}=\left(d_{E}-d_{f}\right)/d_{f}$$ (9)

where $d_{E}$ represents the signaling distance for enhanced signals, $d_{f}$ represents the signaling distance for fast wave signals, $r_{d}$ represents the signaling distance growth ratio comparing the fast wave and enhanced signals.

### 2.6 Statistical analysis

The statistical analysis was performed using MATLAB (2022b) to determine if there are significant differences in the metrics between fast wave and enhanced signals for each subject individually, as well as in an overall analysis combining all subjects. For this comparison, all the high-quality channels were included, and we computed all three metrics for the entire channel without segmentation. Since the differences between paired data are not normally distributed, the data are represented as the median and the interquartile range. A nonparametric Wilcoxon signed-rank test was conducted to determine the significance of the differences observed in the metrics for fast wave and enhanced signals for two related samples. The resulting p-values were compared against a significance level of 0.05 to determine statistical significance.

## 3 Results

### 3.1 Metrics comparison

**FIGURE 7:**
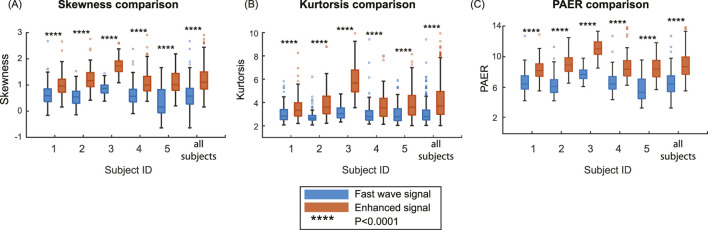
Comparison of the metrics on the group level. **(A)** Skewness comparison between fast wave and enhanced signals. **(B)** Kurtosis comparison between fast wave and enhanced signals. **(C)** PAER comparison between fast wave and enhanced signals.

In this section, we calculated three metrics for all high-quality channels for each subject, as well as conducted an overall analysis by combining the data from all subjects. The boxplots of five subjects and all subjects combined are shown in Figure 7. The median and interquartile range values (IQR) are shown in Table 2. Enhanced signals exhibit significantly higher skewness, kurtosis, and PAER compared to fast wave signals, with all the p-values for five subjects and overall analysis less than 0.0001. Outliers are observed in each metric, and different subjects exhibit variations across all metrics. For example, subject #3 shows noticeably higher values than other subjects in all three metrics for both fast wave and enhanced signals.

**TABLE 2:** Median value and interquartile range values (IQR) of 3 metrics for 5 subjects.

Metrics	Subject #1	Subject #2	Subject #3	Subject #4	Subject #5	All subjects
Skewness Fast wave signal	Median = 0.59 IQR = 0.49	Median = 0.54 IQR = 0.46	Median = 0.87 IQR = 0.30	Median = 0.57 IQR = 0.48	Median = 0.16 IQR = 0.89	Median = 0.58 IQR = 0.60
Skewness Enhanced signal	Median = 0.96 IQR = 0.49	Median = 1.16 IQR = 0.54	Median = 1.73 IQR = 0.40	Median = 1.00 IQR = 0.55	Median = 1.01 IQR = 0.65	Median = 1.11 IQR = 0.65
Kurtosis Fast wave signal	Median = 2.83 IQR = 0.86	Median = 2.64 IQR = 0.39	Median = 3.05 IQR = 0.85	Median = 2.78 IQR = 0.81	Median = 2.77 IQR = 1.02	Median = 2.79 IQR = 0.84
Kurtosis Enhanced signal	Median = 3.34 IQR = 1.13	Median = 3.62 IQR = 1.50	Median = 5.67 IQR = 1.87	Median = 3.55 IQR = 1.54	Median = 3.60 IQR = 1.67	Median = 3.71 IQR = 1.95
PAER Fast wave signal	Median = 6.47 IQR = 1.75	Median = 6.14 IQR = 1.67	Median = 7.71 IQR = 1.12	Median = 6.45 IQR = 1.65	Median = 5.38 IQR = 2.57	Median = 6.47 IQR = 2.08
PAER Enhanced signal	Median = 8.20 IQR = 1.62	Median = 8.93 IQR = 1.77	Median = 11.02 IQR = 1.62	Median = 8.38 IQR = 2.01	Median = 8.40 IQR = 2.15	Median = 8.71 IQR = 2.27

### 3.2 Comparison of uterine contraction detection results between fast wave and enhanced signals

**FIGURE 8:**
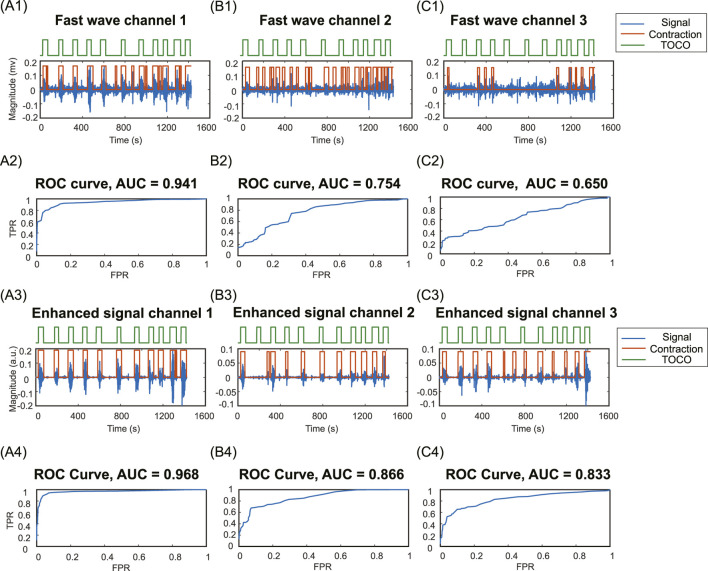
Contraction detection results compared to TOCO. **(A1, B1, C1)** Fast wave signals and contraction detection results with the optimal threshold in channels 1,2, and 3. **(A2, B2, C2)** ROC curves and AUC values for fast wave signals in channels 1,2,3. **(A3, B3, C3)** Enhanced signals and contraction detection results with the optimal threshold in channels 1,2, and 3. **(A4, B4, C4)** ROC curves and AUC values of enhanced signals in channels 1,2,3. TPR, true positive rate; FPR, false positive rate.

Figure 8 illustrates the contraction detection results using the energy-based method. In the channels with high SNR in fast wave signal (Figures 8A1,A2), where the uterine contraction patterns are discernible, the enhanced signal (Figures 8A3,A4) yields marginal improvements in AUC value (from 0.941 to 0.968). However, for fast wave signals with low SNR (Figures 8B1,B2,C1,C2), the enhanced signals (Figures 8B3,B4,C3, C4) remarkably improve the AUC values (from 0.754 to 0.866, from 0.650 to 0.833).

### 3.3 Comparison of signal consistency and signaling distance between fast wave and enhanced signals

For all subjects, the numbers of high consistency channels range from 7 to 50 for fast wave signals and 13 to 55 for enhanced signals. The enhanced signal consistently increases the number of high consistency channels, with the growth ratios ranging from 0.1 to 0.86. Specifically, subject #2 has the lowest growth ratio of 0.1, and subject #4 has the highest growth ratio of 0.86. The number of high-consistency channels for enhanced signals is significantly higher than for fast wave signals, with a p-value less than 0.05 for each group of five subjects.

The enhanced signal consistently increases the signaling distance. The signaling distances range from 77 mm to 166 mm for fast wave signals and from 110 mm to 174 mm for enhanced signals, with the growth ratios ranging from 0.05 to 0.43. Specifically, subject #2 has the lowest increase in signaling distance between the fast wave (166 mm) and the enhanced signal (174 mm). Subject #4 has the largest increase in signaling distance between the fast wave (77 mm) and enhanced signal (110 mm). The signaling distances obtained from enhanced signals are significantly higher than those from fast wave signals, with a p-value less than 0.05 for each group of five subjects. The standard deviation of signaling distance for the enhanced signal (24.62) is lower than that (32.88) for the fast wave signal. The results for each subject are shown in Table 3.

**TABLE 3:** High consistency channel number, growth ratio of channel number, signaling distance, and growth ratio of signaling distance for fast wave and enhanced signals in five subjects.

Signaling metrics	Subject #1	Subject #2	Subject #3	Subject #4	Subject #5	Mean ± std
High consistency channel number Fast wave signal	27	50	24	7	34	28.40 ± 15.6
High consistency channel number Enhanced signal	38	55	28	13	55	37.80 ± 18.05
Growth ratio of channel number	0.41	0.1	0.17	0.86	0.62	0.43 ± 0.32
Signaling distance Fast wave signal	130 mm	166 mm	105 mm	77 mm	125 mm	120.60 ± 32.88
Signaling distance Enhanced signal	148 mm	174 mm	123 mm	110 mm	144 mm	139.80 ± 24.62
Growth ratio of signaling distance	0.14	0.05	0.17	0.43	0.15	0.19 ± 0.14

## 4 Discussion

In this study, our findings suggest that the slow wave signals carry independent and complementary information to improve the contrast between uterine activities and baseline noises. As shown in Figure 7, the enhanced signal exhibits higher values in skewness, kurtosis, and PAER for all subjects. This occurs because the baseline noise component shifted leftward, concentrating closer to zero. Meanwhile, the uterine contraction component for enhanced signal remains largely unchanged, increasing the separation between the uterine contraction and baseline noise. These results suggest that the signal enhancement method effectively preserves the uterine contraction signal while reducing the baseline noise, thereby enhancing signal separability and ensuring a clearer distinction between physiological events and background noise. Despite setting a 95-percentile threshold for the energies to mitigate the effects of spike artifacts, outliers are still observed, as shown in Figure 7. Spike artifacts can significantly increase the skewness, kurtosis, and PAER, as they make energy distribution more skewed, heavier-tailed, and elevate the maximum value. Spike artifacts are very common in the EHG signals. Future studies should focus on developing algorithms to remove spike artifacts or designing more advanced electrodes that are robust to such artifacts to benefit post-processing tasks.

The proposed signal enhancement method offers substantial benefits for uterine contraction detection. The enhanced signals exhibit higher AUC compared to the fast wave signals, as shown in Figure 8. This is due to the high contrast between uterine contraction and baseline noise in enhanced signals, making it easier to find thresholds that effectively separate baseline noise from uterine contractions. By leveraging slow wave signals as an additional source of information for uterine contraction activities, the signal quality can be significantly improved. This enhancement leads to more reliable and noise-resilient contraction detection outcomes. This advancement enhances our understanding of uterine signaling and propagation.

By analyzing the consistency between multi-channel EHG signals and TOCO monitor data, we can evaluate the spatial pattern of uterine contractions. Our results indicate that the number of high consistency channels for enhanced signals is significantly higher than for fast wave signals. Furthermore, the signaling distances estimated using these high consistency channels are significantly higher for enhanced signals compared to fast wave signals. A high signal consistency between multi-channel EHG and TOCO signals suggests high synchronization (Shen et al., 2023; Smith et al., 2015; Young, 2018; 2015) resulting from uterine signaling and propagation. As shown in Figure 6 and Table 3, fast wave signals exhibited high consistency exclusively in channels proximate to the TOCO monitor, likely due to the rapid decay of fast wave, which inherently limits their capability to reflect in long-distance signaling. In contrast, enhanced signals demonstrated a broader spatial distribution in capturing the TOCO signal, suggesting that enhanced signals exhibit greater resilience to attenuation and interference compared to fast wave signals. Therefore, analyzing the spatial pattern of uterine contraction using only fast wave signals tends to underestimate the propagation distance in the laboring uterus. Overall, our data suggest that slow waves are more resistant to noise and carry the detectable contraction signal across a longer distance.

As shown in Table 3, estimating the signaling distance using the enhanced signal results in a lower standard deviation across different subjects compared to the fast wave signal. This indicates that using enhanced signals for estimating signaling distance will lead to more consistent and reliable results. This suggests that the fast wave signal can be easily affected by the experimental conditions, such as background noise levels or the subject’s skin characteristics. In contrast, the enhanced signal is more robust to these experimental conditions, leading to more accurate data analysis.

Our signal enhancement method demonstrated varying efficacy across different subjects, and there are two distinct perspectives to evaluate its benefits: the improvement in signal quality and the improvement in post-processing tasks. From the signal quality perspective, the improvement is primarily influenced by the noise levels in the EHG recordings. The method proved more effective when the original EHG signal has a higher SNR, as this allows clearer identification of fast wave and slow wave signal patterns. For example, subject #3, who has a high-quality EHG signal as shown in Figure 7, exhibited the highest increase in all metrics. However, from the perspective of improvement in the post-processing tasks, if the fast wave already has high SNR, there will be no significant differences in the outcomes of post-processing tasks since there is less room for improvement. For instance, subject #3 does not show a notable increase in the high consistency channel number. Conversely, for low quality fast wave EHG signals, we observed greater benefits in the post-processing tasks. This is because there is more room for improvement when the fast wave signal quality is low.

The selection of a 10-s window was based on physiological and signal processing considerations. Uterine contractions typically last between 30 and 90 s. Therefore, a window size larger than 30 s would oversmooth the signal, making it hard to extract meaningful information from the signal. Conversely, if the window size is too short, the signal would be easily affected by local structures, such as spike artifacts. Additionally, we use the RMS slow wave envelope as the weighting factor to enhance the EHG signal. The frequency band spans from 0.01–0.1 Hz. With a high cutoff frequency of 0.1 Hz, a 10-s sliding window was implemented to effectively capture the information in the slow wave signal.

Our EHG system and signal enhancement can potentially benefit the uterine vector analysis (Garfield et al., 2020). While the prior study positioned the electrode pairs along the X, Y, and Z directions, our use of multi-channel EHG and signal enhancement techniques can enable us to discover more complex and accurate patterns of uterine propagation. In addition, this study may also help distinguish between true labor contractions and Braxton Hicks contractions (Mulder and Visser, 1987), as accurately locating the timing of uterine contractions is a prerequisite for differentiating between the two types. The methodology may prove valuable in analyzing multiple frequency bands of uterine contractions to find the characteristics of labor contractions and Braxton Hicks contractions, which may provide insights into the timing of delivery or the risk of preterm birth (Lucovnik et al., 2011; Maner et al., 2003).

It is important to acknowledge the limitations of this study. First, our method was primarily tested on data obtained from term pregnancies, and its efficacy in preterm birth scenarios requires further investigation. Second, the improvement in accurate contraction detection outcomes was primarily tested using the energy-based method. However, the impact on other contraction detection methods, such as sample entropy (Chen et al., 2024; Shen et al., 2023) or nonlinear correlation (Muszynski et al., 2018), needs further evaluation. Additionally, false positives in contraction detection using the energy-based methods still exist even after signal enhancement. Machine learning (Liu et al., 2015; Yang et al., 2021; Ying et al., 2011) and deep learning methods (El Dine et al., 2022) have undergone significant advancements in recent years, which can potentially be used to further increase contraction detection accuracy. As we extract features from different frequency bands and perform feature fusions to enhance signal quality, more sophisticated feature fusion techniques offered by machine learning and deep learning methods may further improve signal quality. Furthermore, combining the spatial and temporal information from EHG signals can help reduce the effects of background noise and artifacts.

In conclusion, this study presents a novel signal enhancement for uterine contraction detection, leveraging the relationship between slow and fast wave components of EHG signals. This enhancement not only mitigates the impact of noise but also strengthens the physiological relevance of extracted features from EHG signals. Our findings demonstrate significant improvements in signal quality and contraction detection accuracy. Enhanced signal quality could lead to a more comprehensive analysis of spatial signaling patterns of uterine contraction and lay a foundation for the reliable identification of labor onset and progression. As we continue to improve this technique, we anticipate its integration into clinical practice, ultimately contributing to safer and more effective labor management for all pregnancies.

## Data Availability

The sample code and data supporting this study are available at https://github.com/gaohan433/Signal-enhancement.git.
